# Research on the Protrusions Near Silicon-Glass Interface during Cavity Fabrication

**DOI:** 10.3390/mi10060420

**Published:** 2019-06-23

**Authors:** Meng Zhang, Jian Yang, Yurong He, Fan Yang, Yongmei Zhao, Fen Xue, Guowei Han, Chaowei Si, Jin Ning

**Affiliations:** 1 Research Center of Engineering for Semiconductor Integrated Technology, Institute of Semiconductors, Chinese Academy of Sciences, Beijing 100083, China; zhangmeng@semi.ac.cn (M.Z.); yangjian@semi.ac.cn (J.Y.); hyr617@semi.ac.cn (Y.H.); yangfan3104@semi.ac.cn (F.Y.); ymzhao@semi.ac.cn (Y.Z.); 2 Center of Materials Science and Optoelectronics Engineering, University of Chinese Academy of Sciences, Beijing 100049, China; 3 State Key Laboratory of Transducer Technology, Chinese Academy of Sciences, Beijing 100083, China; 4 Department of Electrical Engineering, Stanford University, Stanford, CA 94305, USA; xuefenadvance@gmail.com

**Keywords:** silicon-glass, interface, protrusions, MEMS

## Abstract

Taking advantage of good hermeticity, tiny parasitic capacitance, batch mode fabrication, and compatibility with multiple bonding techniques, the glass-silicon composite substrate manufactured by the glass reflow process has great potential to achieve 3D wafer-level packaging for high performance. However, the difference in etching characteristics between silicon and glass inevitably leads to the formation of the undesired micro-protrusions near the silicon-glass interface when preparing a shallow cavity etched around a few microns in the composite substrate. The micro-protrusions have a comparable height with the depth of the cavity, which increases the risks of damages to sensitive structures and may even trigger electrical breakdown, resulting in thorough device failure. In this paper, we studied the characteristics of the chemical composition and etching mechanisms at the interface carefully and proposed the corresponding optimized solutions that utilized plasma accumulation at the interface to accelerate etching and bridge the gap in etching rates between different chemical compositions. Finally, a smooth transition of 131.1 nm was achieved at the interface, obtaining an ideal etching cavity surface and experimentally demonstrating the feasibility of our proposal. The micromachining solution is beneficial for improving the yield and structural design flexibility of higher performance micro-electromechanical systems (MEMS) devices.

## 1. Introduction

Three-dimensional (3D) wafer-level packaging technology has the great potential to realize smaller size, lower fabrication cost, and high yield [1,2,3] by exploiting wafer bonding with a lid wafer containing vertical electrical feedthroughs. In particular, the vertical feedthrough interconnect technology has been intensively studied owing to its advantages of high integration levels with a small form factor [4], fast speed, and low power consumption due to reduced signal path [5,6], allowing 3D heterogeneous integration [7] for better functionality and process compatibility [3,8]. The fabrication of through wafer vias with high aspect ratio and via filling technologies is the critical process to realize vertical electrical interconnect, which has two important technologies including through silicon via (TSV) and through glass via (TGV). However, in TSV interconnects, additional submicron-thick isolated layer is required to be deposited [9], and the difficulty for this is in the void-free conformal coating of high aspect ratio vias to avoid isolation failure [5,10]. Besides, the signal crosstalk induced by parasitic capacitance coupling between electrical feedthroughs and bulk silicon substrate also limits the application of TSV technology. TGV technology can eliminate the capacitive parasitics owing to the superior insulation property of glass. But the batch fabrication of fine-pitch through glass vias with high aspect ratio and smooth sidewalls is challenging owing to the intrinsic amorphous characteristic of glass. The glass reflow process with a patterned silicon mold was proposed for fabricating a composite substrate where silicon feedthroughs are embedded in the insulation medium of glass, which has a similar coefficient of thermal expansion (CTE) to that of silicon [11]. On account of the advantages of low crosstalk, batch-mode fabrication, and small distortion caused by CTE mismatch, the glass-silicon composite substrate combined with wafer bonding has been applied to the 3D packaging of micro-electromechanical systems (MEMS) pressure sensors [12,13], radio frequency (RF) resonators [14,15,16,17], and gyroscopes [18,19] to obtain good hermeticity, high performance, and high reliability.

Generally, a cavity with a certain depth is necessary to be fabricated in the lid wafer to provide the motion space of fragile sensitive structures and act as the capacitive gap or the reference pressure chamber. Sometimes, non-evaporable getter is even required to be deposited in the cavity to realize and maintain high vacuum for high quality factor [1] and high thermal stability [20,21]. Herein, the glass and silicon in specific regions are required to be etched away respectively to the same depth to form the cavity in the glass-silicon composite substrate. However, in the etched regions where silicon and glass coexist, undesired ridge-like protrusions, which have a comparable height with the depth of the cavity, appear near the silicon-glass interface. The protrusions in the cavity probably impede the motion of sensing structures, which greatly increases the risks of damages to the fragile movable structures. Meanwhile, taking consideration of the narrow air gap between the protrusions and sensing structures, electrical breakdown is prone to be triggered [22], which may lead to the complete failure of the sensors. Consequently, it is imperative to eliminate the protrusions near the silicon-glass interface to achieve high yield and high reliability. 

The reason for the formation of the protrusions has not been reported, and the solution has yet not been provided. The aim of this work is to analyze the formation from two aspects: the differences in the chemical component and the etching mechanism near the silicon-glass interface. Energy dispersive X-ray spectroscopy (EDS) was conducted to determine the chemical composition, and scanning electron microscopy (SEM) was applied to characterize the etching profiles at the silicon-glass interface. The solutions based on the analyses are presented. The feasibility was experimentally demonstrated by the elimination of the undesired micro protrusions, which facilitated the wide application of glass-silicon composite substrate and the realization of complex 3D microsystems with multiple functionalities.

## 2. Experiment Details 

**Figure 1:**
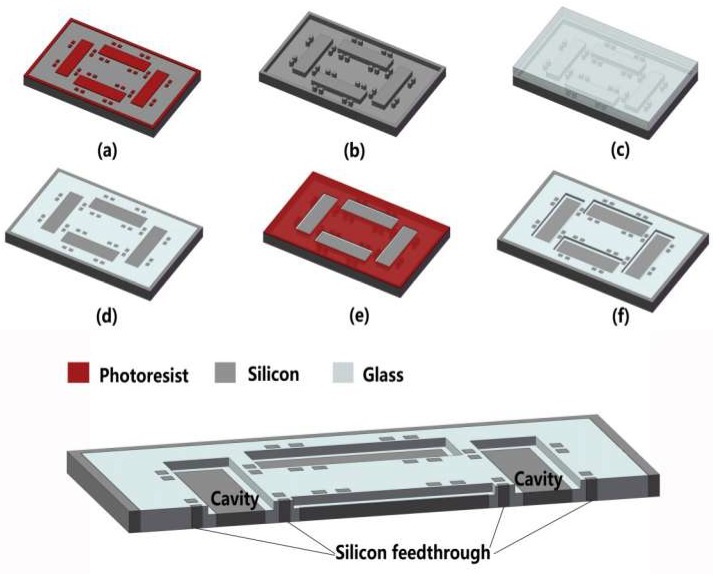
The fabrication flow of glass-silicon composite substrate: (**a**) photolithography for silicon mold; (**b**) silicon deep reactive ion etching; (**c**) anodic bonding between silicon and glass; (**d**) glass reflow and double-side chemical mechanical polishing (CMP) process; (**e**) the cavity defined by photoresist; (**f**) removal of the exposed silicon and glass. The bottom picture depicts a 3D sectional schematic of the glass-silicon composite substrate with the cavity.

The process flow for the silicon-glass composite substrate with cavities is described in Figure 1. Firstly, the photoresist was patterned by standard photolithography technology as the etching mask on a silicon wafer with low electrical resistivity, and then the silicon wafer was etched to the depth of about 300 μm by the deep reactive ion etch (DRIE) process. Afterwards, a Pyrex glass wafer was anodically bonded to the silicon substrate in vacuum in a SUSS SB6e wafer bonder where outgassing was conducted in the meantime. The glass reflow process, based on viscous deformation at the temperature about 1000 °C, which was above the glass softening point of 810 °C, was conducted in a furnace to make the glass refill into the deep silicon trenches. Subsequently, the double-side planarization of the bonded wafer was implemented by chemical mechanical polishing (CMP) technology. 

**Figure 2:**
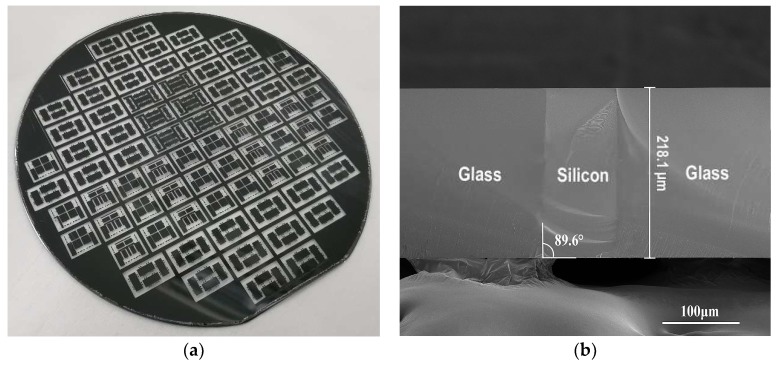
(**a**) Photograph for the glass-silicon composite substrate; (**b**) cross-sectional scanning electron microscopy (SEM) view of the silicon feedthrough after planarization.

The glass-silicon composite substrate and the cross-section view taken by SEM after planarization are exhibited in Figure 2a,b respectively. With the support of mature silicon deep etching technology, fine-pitch silicon vertical feedthrough structures with virtually arbitrary shapes could be fabricated. At the same time, the glass around the silicon vertical feedthroughs provided good insulation to obtain negligible parasitic capacitance compared with the TSV technology. As observed in Figure 2b, a good flatness was still realized by means of the CMP process despite the heterogeneous substrate. A sidewall slope of 89.6° was determined, which was perpendicular to the substrate to avoid the wafer distortion caused by thermal stress gradient of conical holes [2,9]. No void, even interfacial void, was found, which implied good reliability and repeatability. This is another advantage of the vertical interconnect technology based on the glass reflow process over TSV technology.

Finally, a second mask was patterned to define the cavity, which was followed by the removal of exposed silicon and glass to form the desired cavity with smooth inner surfaces; the sectional schematic is displayed at the bottom of Figure 1. The exposed silicon was firstly etched by inductively coupled plasma (ICP) etching technology with fluorine-based gas to acquire smooth etched surfaces. It should be noted that the glass would be slightly etched at the same time where the etching rate of silicon to glass was 15:1. In consideration of the dramatic difference in etching rates of glass and silicon under dry etch conditions, wet chemical etching was adopted for shallow glass etching. Buffered hydrofluoric acid (BHF) solution, containing HF/NH_4_F/DI.H_2_O (deionized water) in a volumetric ratio of 1:2:3, was used for glass etching, while the exposed silicon exhibited chemical inertness. The concentrations of HF and NH_4_F solutions were both 40%. To avoid pinholes generated in the glass surface from the HF solution penetrating through the defects in the mask layer, the metal layers of Cr/Au, with a thickness of 50/100 nm, were deposited to act as the etching mask combined with photoresist [23,24]. Au is chemically inert in HF-based solutions, and the Cr layer was used to increase the adhesion between the Au layer and the surface. They were easily patterned by wet chemical etching after photolithography.

It is worth noting that the local masking effects, originating from insoluble products deposited on the etched surfaces during glass wet etching, decreased the etching rate and increased the surface roughness. Thus, hydrochloric acid (HCl) was added into the solution to remove the insoluble products. As reported in the literature, HF/HCl solutions in a volumetric ratio of 10:1 is optimal for effectively improving etching quality [25]. Therefore, the HF/HCl/DI.H2O solution used at 10:1:30 ratio was also applied for glass etching to serve as a contrast and improve the etching profiles. The concentration of HCl solutions used was 36~38%.

## 3. Results and Discussion

**Figure 3:**
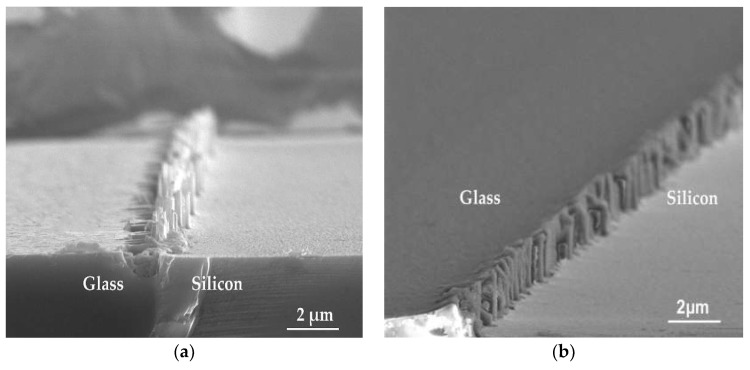
SEM view near glass-silicon interfaces at the bottom surfaces of the cavities after etching. (**a**) Buffered hydrofluoric acid (BHF) for glass etching, and etching time was 8 min; (**b**) HF/HCl/DI.H2O (deionized water) solution for glass etching, and etching time was 1 minute.

The exposed silicon and glass were etched to the same depth of 2 μm in sequence to fabricate the designed cavities that were expected to have smooth etching surfaces. In reality, however, the etching profiles of the cavity, especially near the silicon-glass interfaces, did not conform to the expectations due to the complex interactive mechanisms, which were taken by SEM as shown in Figure 3. Figure 3a shows the bottom etching surface of the cavity where the glass was etched by BHF solution for 8 min, while in Figure 3b, the glass was etched by HF solution with added HCl for 1 min to the same depth of 2 μm. Obviously, the addition of HCl into the HF solution indeed increased the etching rate of glass by transforming the insoluble products to be soluble. In addition, prolonged HF etching resulted in the formation of the valleys in the glass region adjacent to the silicon-glass interface as shown in Figure 3a. Therefore, the HF solution with added HCl is more appropriate for glass etching in these conditions. 

Special attention has been paid to the protrusions that occurred near the silicon-glass interfaces shown in Figure 3. The heights of the protrusions were about 1.2 μm as measured by Stylus Profiler, which was comparable with the depth of the cavity and became the potential safety hazard to the fragile sensing structures in the following device fabrication. To eliminate the protrusions, the chemical composition and the reaction mechanisms near the interface different from other regions were investigated.

### 3.1. Chemical Composition

**Figure 4:**
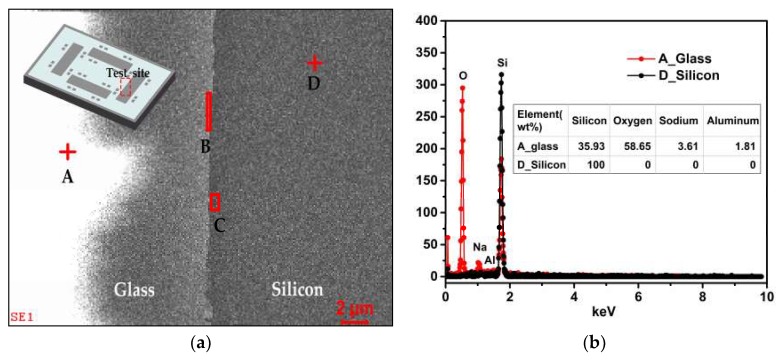
(**a**) The distribution of four test regions for energy dispersive X-ray spectroscopy (EDS) measurement; (**b**) the energy spectrums and relative content of each element in weight percentage measured for areas A and D.

Firstly, the chemical compositions of the samples were measured by energy dispersive X-ray spectroscopy (EDS). The characteristic X-ray of each chemical element is unique. As a result, the chemical elements and their relative contents in the samples can be reflected qualitatively by the strength of the corresponding X-ray in the energy spectrum. For the sake of contrast, four regions were taken respectively from glass to silicon, and the distributions are indicated in Figure 4a. The measurement site was taken on the polished surface after the CMP process as shown in Figure 4a. Regions A and D were located in reflowed glass and silicon regions, respectively. The transition areas B and C were situated approximately beside the silicon-glass interface in an opposing manner. 

Figure 4b shows the energy spectrums measured for regions A and D, and the relative content of each element in weight percentage is also listed. Obviously, only one element of silicon was contained in silicon area A, while in glass area D, sodium, aluminum, and oxygen coexisted with silicon. The measurement results are consist with the practice [26,27], and the validity of chemical compositions determined by EDS is certified.

To quantitatively evaluate the differences in chemical composition among four regions, the relative contents for each element contained were acquired by EDS measurement in the form of weight percent (wt%). To minimize the test error, multiple different testing points were taken in four regions A, B, C, and D, and the averages were calculated and listed in Table 1. In the silicon region D, the weight percent of silicon element was 100%, which implied only silicon existed there. It was also clearly indicated that three elements of sodium, aluminum, and oxygen were also contained in addition to silicon in areas B and C near the interface, which was similar with the glass area A. 

**Figure 5:**
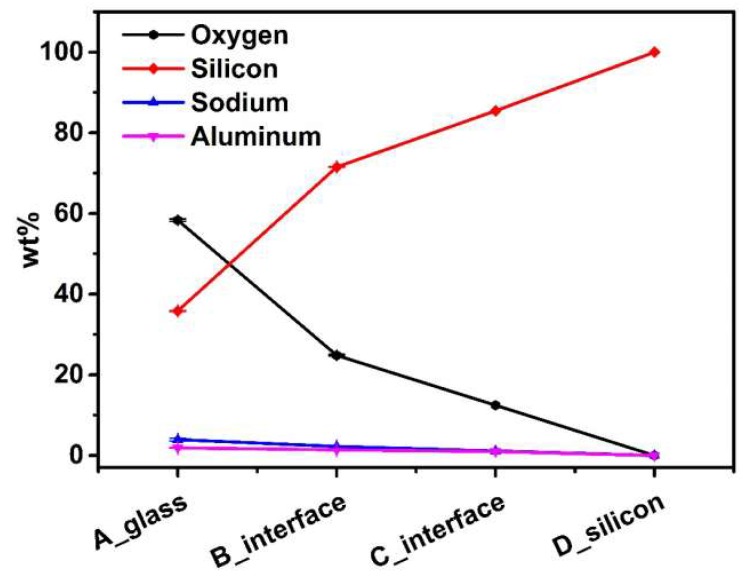
The chemical composition contained in the four test regions in weight percentage (wt%).

To vividly illustrate the differences in chemical components among the four regions, the data in Table 1 is plotted and exhibited in Figure 5. In comparison with the glass area A, the relative contents of oxygen were lower in areas B and C near the interface, which was related to the anodic bonding process and glass reflow process. Under a high voltage electrostatic field, the movable metal ions in glass migrate to the cathode to form the depletion zone near the interface [28,29]. The oxygen anions migrate from glass to the silicon to form Si-O bond at the bonding interface, while in the cavities, the oxygen exists in the form of gaseous state in particular [30,31,32]. The generation of O_2_ during the anodic bonding and thermal oxidation occurring to the sidewalls of the silicon deep trenches during the glass reflow process at a high temperature of about 1000 °C should be responsible for the absence of oxygen in area C, which was located mainly in the silicon region. Because the thermally grown oxide layer on silicon sidewalls was thin, the relative contents of oxygen in area C were much lower than in the reflowed glass area A.

In terms of the particularity in the chemical composition of the silicon-glass interface, the concluded reason for the formation of the protrusions near the silicon-glass interface is that the SiO_2_ layer on the silicon sidewalls—due to thermal oxidation during the glass reflow process—was etched very slowly in both the etching processes of silicon and glass. This is why the protrusions were mainly located in the silicon region. On the one hand, under ICP etching conditions, the thermally grown SiO_2_ was etched much slower than silicon. On the other hand, with the HF solution, the glass was etched about five times faster than thermally grown oxide layer [33]. As a result, the material near the silicon-glass interface still remained to form the micro protrusions while the materials in other regions were etched away. 

### 3.2. Etching Mechanisms

**Figure 6:**
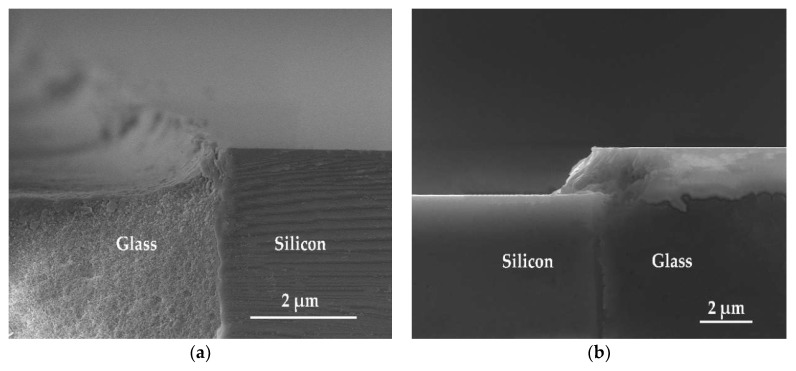
SEM cross-sectional views for silicon-glass interfaces. (**a**) Only glass was etched by HF-based solution with added HCl; (**b**) only silicon was etched by inductively coupled plasma (ICP).

The difference in reaction mechanisms near the silicon-glass interface from other regions was also studied. Samples where (1) only glass was etched by an HF-based solution with added HCl and (2) only silicon was etched by the ICP process were prepared. The cross-sectional views of silicon-glass interfaces taken by SEM are given in Figure 6 where it can be observed that the etched sidewalls are both not perpendicular, and the materials unremoved near the interface increases gradually from top to bottom. The curved etching sidewalls of glass in Figure 6a is normal and arises from the isotropic nature of wet etching [27]. The rim of the top opening was limited by silicon edges at the interface owing to the shield of silicon.

**Figure 7:**
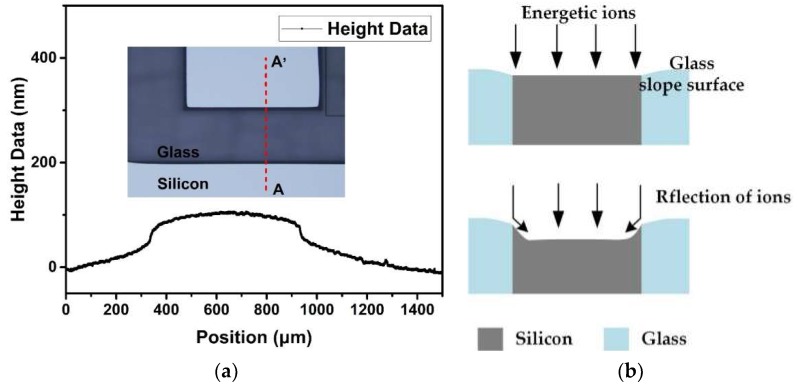
(**a**) The profile measured by Stylus Profiler from point A to A’; (**b**) the model for silicon etched profiles, which was influenced by the slope glass surface.

The reason for the declined sidewall after silicon etching in Figure 6b can be explained with two aspects: For one, the oxide layer—due to thermal oxidation—is inherently etched more slowly than silicon; for another, the base angle of etch profiles depends on the mask profiles [34]. It should be noted that the glass was higher than silicon after planarization by adjusting the polishing parameters and the composition of polishing slurry to ensure the close contact between the glass bonding region and the silicon wafer prefabricated with sensitive structures in the following bonding process. The height of the step was about 80 nm as measured by Stylus Profiler, as shown in Figure 7a, and the transition between glass and silicon was gentle rather than steep, which meant that the part of glass higher than silicon would work as a tapered mask for silicon etching, as shown in Figure 7b. The reflection of ions on the glass slope sidewalls resulted in the declined silicon etching sidewall. 

In conclusion, the formation of protrusions is attributed to the oxide layer grown by thermal oxidation during the glass reflow process and the mutual shielding effect between glass and silicon during etching. The protrusions near the interface in Figure 3a consisted of remaining glass, owing to the isotropic nature of glass wet etching, remaining silicon on account of ion reflection on the glass slope, and the thermally grown SiO_2_ layer during the glass reflow process. As a result, minimizing the mutual influence between glass and silicon and increasing the etching rate of the materials near the interface were both necessary to eliminate the protrusions near the silicon-glass interfaces.

## 4. Optimized Fabrication Scheme

Firstly, to avoid the influence of the glass slopes to silicon during ICP etching, glass that was higher than silicon was required to be etched away in advance. Secondly, to break the limit of the silicon edge to glass, the silicon at the interface was removed thoroughly by the isotropic dry etching process before glass etching. In the optimized scheme, the etching of glass alternated with the etching of silicon to minimize the mutual impacts on each other.

Furthermore, the whole etching process was broken up into multiple parts to be conducted to obtain smaller protrusions. Furthermore, the protrusions were completely exposed to the ICP etching condition and etched more than once, which could bridge the gap in etching rates of the thermally grown oxide and glass. As a result, the protrusions could be eliminated step by step. 

**Figure 8:**
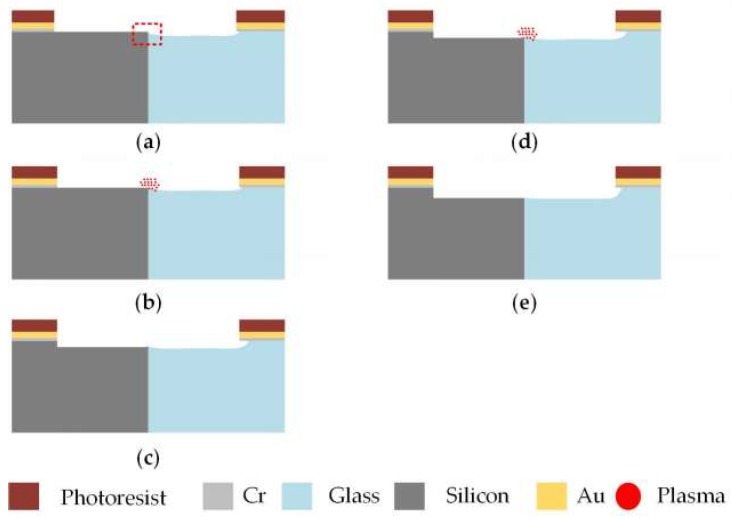
Optimized process flow for fabricating a 2 μm cavity. (**a**) The glass was etched by HF-based solution with added HCl for a few seconds; (**b**) the silicon was etched to 1 μm using ICP etching technology; (**c**) the glass was also etched to be 1 μm; (**d**) the silicon was etched to 2 μm, as in step (**b**); and (**e**) the glass was also etched, as in step (**c**), to the same depth to form a 2-μm-deep cavity.

The optimized fabrication scheme is put forward as shown in Figure 8, which takes the fabrication of a 2 μm-deep cavity as an example. The cavity was broken up into two parts to minimize the size of the protrusions and accelerate the etching of thermally grown oxide by experiencing ICP etching twice.

(a) At first, the sample was immersed in the HF-based solution with added HCl for 5 s to remove the part of glass about 80 nm higher than silicon to avoid the effect of ion reflection on the glass slope. The oxide layer thermally grown on the silicon sidewalls was simultaneously exposed.
(b) Then, the silicon was etched for 24 s to the depth of 1 μm by ICP dry etching technology. During etching, the plasma groups consisting of energetic ions accumulated at the interface to accelerate the etching of the oxide layer. Since plasma accumulation accelerated etching and the ion reflection at glass edges attenuated, much less material remained near the interface to form the protrusions.
(c) Afterwards, without the shield effect of silicon at the interface, 1 μm glass was etched completely, which exposed the protrusions near the interface completely.
(d) One micrometer of silicon was etched away again, as in step (b). During the silicon etching process, the protrusions that were completely exposed were etched more quickly than before in order to be minimized further.
(e) Finally, the glass was also etched to same depth as silicon, as in step (c). By this time, a 2 μm-deep cavity with tiny protrusions was prepared. It should be mentioned that 2-min BHF wet etching could be adopted to further make the step between glass and silicon become smoother. Cavity with other depths could be fabricated by adjusting the number of the etching steps and the etching depth in each step in the optimized process flow in Figure 8.

**Figure 9:**
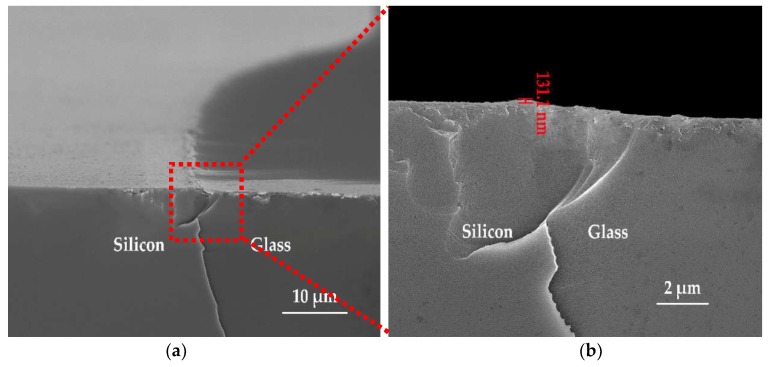
SEM cross-sectional view of the silicon-glass interface prepared with the optimized scheme. (**a**) Silicon-glass interface taken with 5000× magnification; (**b**) close-up view of the silicon-glass interface in (**a**).

The silicon-glass interface of the cavity prepared with the above optimized scheme was observed by SEM and shown in Figure 9. From Figure 9a, it can be obviously observed that the protrusions were essentially removed, and a gentle transition between silicon and glass was obtained. The close-up view for the interface is shown in Figure 9b. The step at the silicon-glass interface was measured as 131.1 nm, which was small enough to avoid the risks of damages to the sensing structures and electrical breakdown owing to a narrow air gap. The feasibility of the optimized etching scheme and the rationality of the analyses were experimentally validated.

## 5. Conclusions

The difference in etching rates between different chemical compositions and the mutual influences between glass and silicon during the etching process results in the formation of protrusions with a comparable height with the depth of cavity near the silicon-glass interface. The protrusions are detrimental for the fabrication of MEMS sensors, which likely causes damage to the fragile sensitive structure and even trigger electrical breakdown due to the extremely narrow air gap. An optimized scheme was proposed based on the analysis from chemical compositions and etching mechanisms. Glass etching and silicon etching were carried out alternatively to minimize the mutual effects on each other. At the same time, breaking up the whole etching into parts minimized the size of the protrusions, and the energetic ion accumulation at the interface accelerated the etching of protrusions, which could bridge the gap in etching rates. The feasibility of the optimized scheme was experimentally demonstrated by obtaining a gentle transition with a step of 131.1 nm at the silicon-glass interface. The elimination of protrusions is beneficial to improve yield and reliability, which facilitates the extensive application of the composite substrate based on the glass reflow process to achieve a 3D MEMS package with high performance. Furthermore, the mechanisms can be extended to other composite substrates to realize a 3D microsystems with more functionalities.

## Figures and Tables

**Table 1:** The average weight percent (wt%) for each element in four various regions.

		Element	Oxygen	Silicon	Sodium	Aluminum
	wt%					
Location						
A_glass			58.335	35.83	3.94	1.895
B_interface			19.48	77.027	2.01	1.483
C_interface			12.45	85.45	1.12	0.98
D_silicon			0	100	0	0
